# Stretching Fibroblasts Remodels Fibronectin and Alters Cancer Cell Migration

**DOI:** 10.1038/srep08334

**Published:** 2015-02-09

**Authors:** Mingfang Ao, Bryson M. Brewer, Lijie Yang, Omar E. Franco Coronel, Simon W. Hayward, Donna J. Webb, Deyu Li

**Affiliations:** 1 Department of Biological Sciences, Vanderbilt University, Nashville, TN; 2 Department of Mechanical Engineering, Vanderbilt University, Nashville, TN; 3 Department of Cancer Biology, Vanderbilt University, Nashville, TN; 4 Department of Urologic Surgery, Vanderbilt University, Nashville, TN

## Abstract

Most investigations of cancer-stroma interactions have focused on biochemical signaling effects, with much less attention being paid to biophysical factors. In this study, we investigated the role of mechanical stimuli on human prostatic fibroblasts using a microfluidic platform that was adapted for our experiments and further developed for both repeatable performance among multiple assays and for compatibility with high-resolution confocal microscopy. Results show that mechanical stretching of normal tissue-associated fibroblasts (NAFs) alters the structure of secreted fibronectin. Specifically, unstretched NAFs deposit and assemble fibronectin in a random, mesh-like arrangement, while stretched NAFs produce matrix with a more organized, linearly aligned structure. Moreover, the stretched NAFs exhibited an enhanced capability for directing co-cultured cancer cell migration in a persistent manner. Furthermore, we show that stretching NAFs triggers complex biochemical signaling events through the observation of increased expression of platelet derived growth factor receptor α (PDGFRα). A comparison of these behaviors with those of cancer-associated fibroblasts (CAFs) indicates that the observed phenotypes of stretched NAFs are similar to those associated with CAFs, suggesting that mechanical stress is a critical factor in NAF activation and CAF genesis.

Although most cancers, and by definition all carcinomas, are derived from epithelia, interactions with other cell types in the surrounding host microenvironment (stroma) are crucial for tumor cell growth, survival, and metastatic spread^1^,^2^. For example, cancer-associated fibroblasts (CAFs) have been shown to induce and promote the invasion of initiated but non-malignant epithelium^3^, as well as cancer cells raised from the human colon^4^, breast^5^, prostate^6^, and other organs. To date, most of these studies have focused on cancer-stroma interactions via paracrine biochemical mediation. In contrast, the role of mechanical stress in cancer cell invasion has not attracted much attention, even though mechanotransduction has been shown to be important in fibroblast functions such as the production and remodeling of extracellular matrix (ECM) components^7^,^8^.

The study of mechanical stress-induced fibroblastic responses can be traced back more than three decades. The pioneering work was conducted both *in vitro* and *in vivo* to examine periodontal ligament alteration upon exposure to mechanical forces^9^,^10^. As the dominant cell type in the periodontal ligament, fibroblasts sense and react to altered mechanical environments by modulating the synthesis and fibrillar assembly of ECM components, including collagen and fibronectin^11^,^12^. These observations in dentistry soon drew attention from the cardiovascular field in which mechanical stress is associated with cardiac dysfunction in hypertensive heart diseases. Increased mechanical stress was shown to cause myocardial hypertrophy, vessel wall thickening, and fibrosis^13^. An additional study showed that a 10% uniaxial stretch of cardiac fibroblasts resulted in an increased expression of fibronectin and collagen type III mRNA. Interestingly, when a 20% stretch was applied, mean levels of fibronectin and collagen III mRNA were lower than the unstretched control^14^. Similar force magnitude dependence was also found in trials of equibiaxial stretching^15^.

The reorientation of fibroblasts under uniaxial cyclic stretch has also been a subject of research. Stretched fibroblasts were reported to migrate faster and farther than their unstretched counterparts while also aligning themselves perpendicular to the direction of stretching^16^. Fibroblast morphology is also affected by the application of mechanical stress. For example, a study that examined the stretching of whole tissues both *in vivo* and *ex vivo* found that fibroblasts in the stretched tissues exhibited larger, “sheetlike” cell bodies with shorter processes, while fibroblasts in unstretched tissues demonstrated a more dendritic morphology with smaller, more globular cell bodies and longer processes^17^.

Mechanical force is also considered to be an important factor in the formation of activated fibroblasts called myofibroblasts (MFs)^18^. Characterized by their contractile activity, MFs are commonly found at injury sites, where their primary function is to close the wound^19^,^20^. These cells are also an important component of the reactive stroma found at sites of chronic inflammation, fibrosis, and surrounding a variety of malignancies^21^,^22^,^23^. In fact, many myofibroblast characteristics, such as smooth muscle actin (SMA) expression, the acquisition of contractile stress fibers, *de novo* expression of the extra domain-A (ED-A) alternative splice variant of fibronectin, the formation of cell-cell contacts through gap junctions^24^, and the secretion of higher levels of cytokines and chemokines are also observed in another population of activated fibroblasts referred to as CAFs^25^. Heterogeneous populations of CAFs are found in abundance in the stroma of numerous tumors, including breast, prostate, and pancreatic carcinomas^2^. It is also worth noting that although CAFs were once thought of as a discrete population, it is now becoming clear that the CAF phenotype in the tumor microenvironment represents a downstream consequence of the interactions among multiple fibroblastic populations^26^,^27^. Nonetheless, both MFs and CAFs alter the production, digestion, and organization of the ECM^28^, and these properties have been shown to contribute to CAFs' role in the promotion of cancer invasion and metastasis^2^,^29^,^30^. The phenotypic similarities between these two cell types suggests that, just as it is for MFs, mechanical force may also be an important factor in the formation of CAFs.

Although a multitude of studies have attempted to characterize CAF gene expression, signaling pathways, and tumor promoting functions, the mechanisms by which CAFs are actually generated in tumors are still not well understood; however, NAFs are thought to be one of the major sources for CAFs^31^,^32^,^33^. Surprisingly, no work has yet demonstrated the direct influence of mechanical stress in CAF genesis, although a growing body of evidence suggests that mechanical changes in the tumor microenvironment are induced by the expansion of tumor cells. As a tumor grows, it exerts forces on the surrounding tissue, inducing local compressive stresses (in the direction of tumor growth) and circumferential tensile stresses^34^. These forces could have important effects on the non-tumor cells in the microenvironment.

Considering the fact that mechanical stress exists in the tumor microenvironment and is an important factor in many fibroblast activities, it is possible that mechanical stress plays a role in the activation of fibroblasts, resulting in the formation of CAFs and leading to ECM remodeling of the tumor microenvironment and the promotion of cancer cell migration. To test this hypothesis, we used a microfluidic platform to stretch fibroblasts derived from normal tissues (NAFs) and observed the responses to the external mechanical stimulus. We show here that mechanical stretching can modify NAF-produced fibronectin alignment and stimulate the fibroblasts' ability to direct cancer cell migration in a persistent way. These data suggest that mechanical stress plays a significant role in the genesis of CAF phenotypes and thus may contribute to the subsequent promotion of cancer progression.

## Results and Discussion

### Microfluidic Stretcher Design and Fabrication

**Figure 1:**
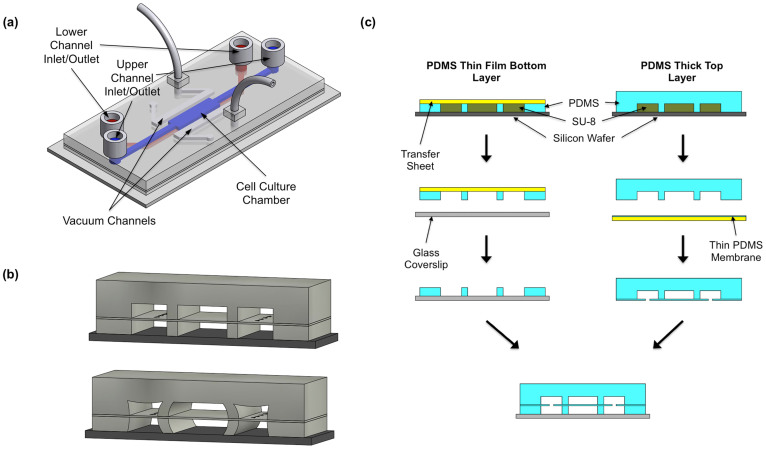
Schematic of a microfluidic stretcher device and its fabrication. (a) A three-dimensional schematic (not to scale) of the stretcher platform showcases the key device features. (b) A cross-sectional view of the device in the relaxed state (top) and stretched state (bottom) after a vacuum has been applied in the outer chambers. (c) The fabrication of the device involves constructing a bottom thin-film layer, a thick top layer, and a thin membrane. These components are assembled using plasma bonding and reside on a thin glass coverslip substrate.

The microfluidic stretcher platform used in this study is based on the work by Huh et al., which developed a microdevice capable of reconstituting the alveolar-capillary interface of the human lung^35^. A three-dimensional schematic of the device is shown in Fig. 1a. Applying a negative pressure to the two vacuum channels on each side of a middle chamber with a suspended PDMS membrane causes the side walls of the middle chamber to deflect outward, imparting a mechanical stretch on cells attached to the suspended PDMS membrane (Fig. 1b). Both the top and bottom layers of the central cell culture chamber are 5 mm in length (*l*), 1 mm wide (*w*), and 100 μm in height (*h*). On the other hand, the inlet/outlet channels for the central chamber are only 400 μm wide. The wider cell culture chamber serves to reduce the flow velocity in that region, which helps to slow down cells that have been loaded through the inlet reservoir and facilitate attachment of the cells to the suspended PDMS membrane (10 μm thick). Each layer of the vacuum channel region is 550 μm (*w*) × 100 μm (*h*) and spans the length of the cell culture chamber region. Finally, the thickness of the wall between the outer vacuum channels and the central cell culture chamber is 75 μm. It was determined through trial and error that this wall thickness allowed for easy manual alignment of the top and bottom layers during fabrication while still yielding desired membrane strains.

Several significant modifications were made to the original microfluidic stretcher design outlined by Huh et al. to simplify fabrication, enhance functionality, and improve reproducibility. First, no etching of the PDMS membrane in the vacuum channels was performed. The etchant used to etch PDMS membranes, a mixture of tetrabutylammonium fluoride (TBAF) and N-methylpyrrolidinone (NMP), is highly flammable, can cause severe skin burns and eye irritation, and is carcinogenic^36^, which makes working with the chemical less than ideal. More importantly, it is difficult to control the final thickness of the channel walls from device to device, as the PDMS etching rate is relatively high (~5 μm/minute) and etchant loading and removal must occur through the inlet and outlet reservoirs. This becomes problematic when consistent strains need to be applied in multiple assays using different devices, as the deflection of the PDMS sidewalls is inversely proportional to the thickness cubed. Thus, small changes in channel wall thickness could result in significantly different membrane strains for a given applied vacuum pressure. For our devices, instead of etching the PDMS membrane, small holes were punched in the suspended membrane in each vacuum channel using a 30-gauge needle. This process connects the top and bottom channels, which yields uniform vacuum pressure in the vacuum channel while improving the consistency among devices. This is because without the etching step that is difficult to precisely control, only the dimensions of the SU-8 mold govern the thickness of the channel sidewalls. Note that the punching is only performed in the vacuum channels on each side, and in the cell culture chamber, we have a solid PDMS membrane as opposed to the porous membrane employed by Huh et al. Unlike the “lung-on-a-chip” device, our microfluidic platform does not require any permeability between cells cultured on the top and bottom of the suspended membrane; thus, we chose to use the simple solid, stretchable PDMS membrane for the application of mechanical stress to fibroblasts cultured only on top of the membrane. For the second modification, an open channel thin PDMS film was used as the bottom layer of the device as opposed to a thick PDMS piece identical to that used for the top layer of the device (see Fig. 1c). Combined with a thin glass coverslip, the 100 μm thick bottom layer renders the device more compatible with advanced confocal microscopy and live-cell imaging. Additionally, this fabrication technique allows for the detachment of the top layer/thin membrane assembly after device use and subsequently enabled imaging of fixed cells at even higher magnifications, which is extremely important for examination of fine structures such as the alignment of secreted fibronectin fibers.

### Device Performance and Characterization

**Figure 2:**
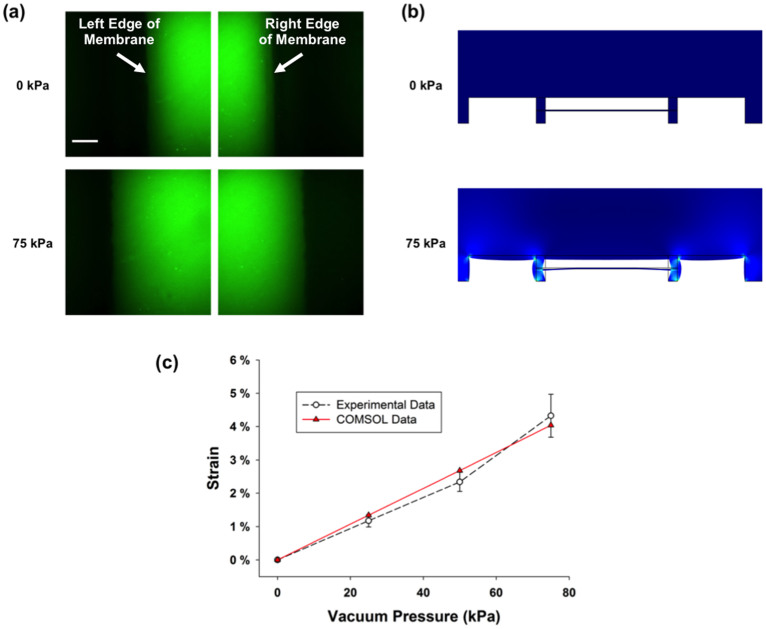
Experimental and FEM characterization of the microfluidic stretcher platform. (a) Characteristic images of the top of the PDMS membrane under different applied vacuum pressures obtained using confocal microscopy. The scale bar represents 20 μm. (b) Visualization of the displacement of the PDMS membrane and sidewalls at different vacuum pressures determined using COMSOL Multiphysics™. (c) A scatter plot compares the strains observed at different vacuum pressures using both experimental and modeling characterization techniques. The error bars associated with the experimental data represent standard deviation. Five separate devices were tested to generate the experimental data.

Both the performance of the stretcher platform and device-to-device consistency were investigated using confocal microscopy. Stacks of images were obtained at the left and right edges of the central cell culture chamber for different applied vacuum pressures. The image within the stack that represented the top of the PDMS membrane was determined by analyzing orthogonal reconstructions of the stack. Then, the displacement of the edge of the membrane, as vacuum pressures were applied, could be measured. Examples of these confocal microscopy images are shown in Fig. 2a. To characterize the performance, a total of five devices were tested, and the measured strains are shown in Fig. 2c. As demonstrated by the error bars in this plot (representing standard deviation), the strains observed among the five devices were reasonably consistent, which indicates that our fabrication technique of punching holes to balance the vacuum pressures in the upper and lower vacuum channels is an effective approach for constructing stretcher devices with reproducible performance.

In addition to the experimental investigation performed, the stretcher device was also characterized using FEM with COMSOL Multiphysics™. A two-dimensional model was constructed with the identical geometry of the stretcher device, and the displacement was calculated for various applied vacuum pressures, as shown in Fig. 2b. The extracted strain from the modeling, plotted in Fig. 2c, agrees well with the experimental data obtained using confocal microscopy. Therefore, this two-dimensional model with low computational requirements could be an effective tool for designing future stretchers with different strain and dimensional requirements.

### Effect of Mechanical Stimulus on Fibronectin Organization of Normal Fibroblasts (NAFs)

**Figure 3:**
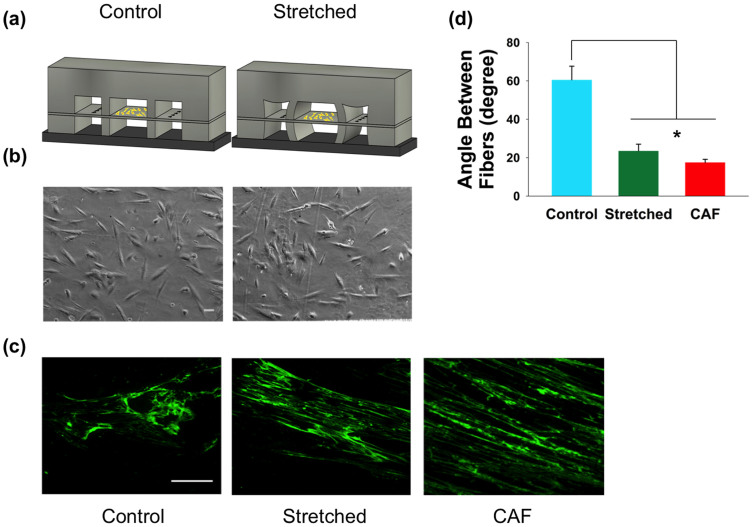
Stretched NAFs deposit fibronectin in an organized format. (a) NAFs (yellow) were grown in control (unstretched) and stretched devices. (b) Representative phase images of NAFs grown in control or stretched devices (after stretching), illustrating the random orientation of the cells attached to the PDMS membrane (scale bar is 50 μm). (c) Characteristic images demonstrating the immunostaining of fibronectin produced by unstretched NAFs, stretched NAFs, as well as CAFs (scale bar is 50 μm). (d) Average angles between fibronectin fibers measured as reported^56^ to compare fibronectin alignment. Both CAFs and stretched NAFs produced fibronectin with smaller angles than the fibronectin produced by unstretched NAFs. Error bars represent S.E.M for 50 cells from three individual experiments (*p < 0.002).

To observe the effects of mechanical force in the activation of fibroblasts, we first examined the fibronectin fibers produced by human prostatic NAFs. We employed the microfluidic stretcher device to exert mechanical force on NAFs by loading the cells onto the suspended membrane and then stretching it (Fig. 3a). NAFs cultured on the PDMS membrane were stretched overnight under a consistent strain of ~4%, after which the stress was released and the NAFs were incubated for another ~24 hours before immunofluorescence staining was performed. Incubation for an additional ~24 hours was necessary to allow the fibroblasts sufficient time to deposit and assemble enough fibronectin for clear imaging. Note that even though the extra 24 hour incubation period was performed after the stretch was released, the fibronectin organization discussed below was still observable immediately after releasing the stretch, indicating that it was the stretching mechanical stimulus that initiated the phenotypic change, not the removal of the strain.

Organization of the fibronectin produced by the NAFs that had been stretched was noticeably different from the fibronectin organization of the control, i.e., unstretched NAFs. The fibronectin produced by unstretched NAFs formed a random network, while the fibronectin secreted by stretched NAFs formed a more aligned, linear network (Fig. 3c). As a comparison, we also examined the fibronectin produced by prostatic CAFs (unstretched) and found that the alignment of CAF-produced fibronectin was similar to that produced by stretched NAFs (Fig. 3c). To quantitatively characterize the fibronectin alignment, we calculated the average angle between fibronectin fibers. The results in Fig. 3d reveal that the average angle between fibronectin fibers produced by unstretched NAFs is 60.5°, whereas the angle between fibronectin fibers produced by stretched NAFs was 23.5°, which is comparable to the 17.5° average measured for CAFs.

It is worth noting that the fibronectin alignment observed in stretched NAFs was not along the stretch direction, but rather along the long axis of the fibroblasts themselves, which were oriented in a random manner (Fig. 3b). This behavior is different from previous studies showing that fibroblasts exhibit a global alignment perpendicular to the stretch direction^16^,^37^. Most likely, the lack of global alignment could be attributed to two reasons. First, the magnitude of strain applied in our experiments is less than that used for previous investigations^16^. As discussed earlier, varying stretch magnitudes may induce different fibroblast responses^14^. Second, a sustained, constant stretch was applied to NAFs in our experiment. In contrast, observations of global fibroblast alignment typically occur upon the application of cyclical stretching^14^,^15^,^38^,^39^,^40^,^41^. Thus, both the magnitude and the manner of exerting the stretch may produce different global fibroblast responses. Note that in these experiments, neither immediately after releasing the stretch nor after the 24 hour post-stretch incubation period was global alignment observed. Nonetheless, these results suggest that the altered fibronectin morphology produced by stretched NAFs is not a simple realignment of the fibronectin fibers under the applied mechanical strain to the substrate, but rather a consequence of a mechanical stimulus causing a fundamental biological change in individual fibroblasts.

### Directed Cancer Cell Migration with Stretched NAFs

**Figure 4:**
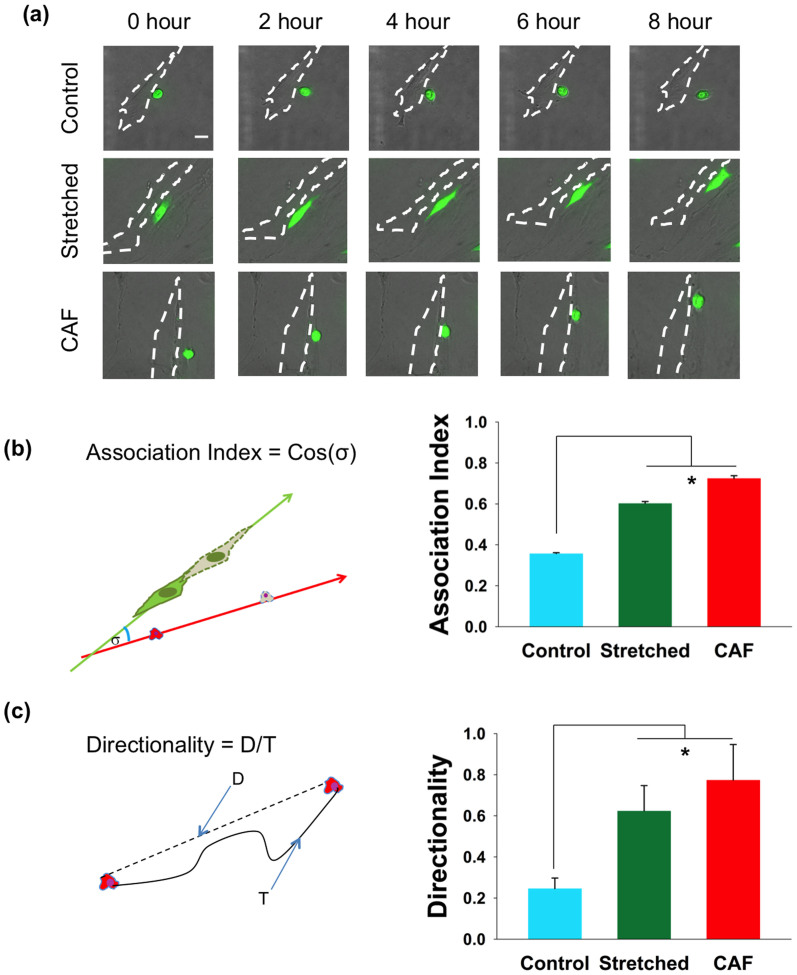
Stretched NAFs direct the migration of cancer cells. SCC61 cancer cells (green) were co-cultured with NAFs and CAFs, whose contour is shown with white dashed lines. (a) Time-lapse images show SCC61 cells move randomly when co-cultured with unstretched NAFs (upper panel), but they move along stretched NAFs and migrate in a persistent way (middle panel) similar to SCC61 cells co-cultured with CAFs (lower panel). The scale bar is 50 μm. (b) Quantification of the association index shows a significant difference when SCC61 cells are co-cultured with stretched NAFs or CAFs vs. unstretched NAFs. Error bars represent S.E.M from 40 cells from six individual experiments (*p < 0.002). (c) SCC61 cells show a higher directionality migration index when co-cultured with stretched NAFs or CAFs compared with unstretched NAFs. Error bars represent S.E.M from 40 cells from six individual experiments (*p < 0.001).

The above study of fibronectin morphology indicates that a mechanical stimulus activates NAFs in such a way as to induce more CAF-like fibronectin organization. Concurrently, it is known that modified ECM in the tumor microenvironment facilitates cancer cell migration^42^. That is, cancer cells tend to migrate in the aligned direction of modified ECM. Thus, it is reasonable to ask whether cancer cells co-cultured with stretched NAFs migrate in a manner similar to cancer cells co-cultured with CAFs. To answer this question, we co-cultured cancer cells with stretched and unstretched (control) NAFs, as well as CAFs, and assessed cancer cell migration for each case. A co-culture was set up by introducing equal amounts of SCC61 head and neck squamous carcinoma cells into the devices (after the overnight stretching period for the stretched NAFs trial). From time-lapse microscopy images (and movies in supplementary information), SCC61 cells in the stretched device appeared to migrate toward and along the NAFs (Fig. 4a, middle panel), which is similar to that observed in a co-culture of SCC61 cells and CAFs (Fig. 4a, lower panel). In contrast, SCC61 cells do not migrate toward unstretched NAFs in the control experiment (Fig. 4a, upper panel). These results suggest that stretching alters the influence of NAFs on the migration of cancer cells. Indeed, quantification shows that SCC61 cells have a higher association index (the cosine of the angle between the migration axes of the fibroblast and SCC61 cells) with stretched NAFs and CAFs when compared with unstretched NAFs (Fig. 4b). Moreover, the directional migration index (directionality), which is the ratio of the net (D) to total distance migrated (T), is significantly higher for SCC61 cells cultured with stretched NAFs and CAFs (Fig. 4c). Since one important function of CAFs is to facilitate cancer cell migration, the similarity of cancer cell migration among stretched NAFs and CAFs indicate that stretched NAFs display an important CAF phenotype, which is in contrast to unstretched NAFs. This further underscores the importance of mechanical stress in NAF activation. It is worth noting that the directional migration could be closely related to the aligned fibronectin secreted by stretched NAFs and CAFs, as it has been suggested that the well-organized fibers in the ECM of cancers help promote cancer metastasis^43^.

### PDGFRα Expression in Stretched NAFs

**Figure 5:**
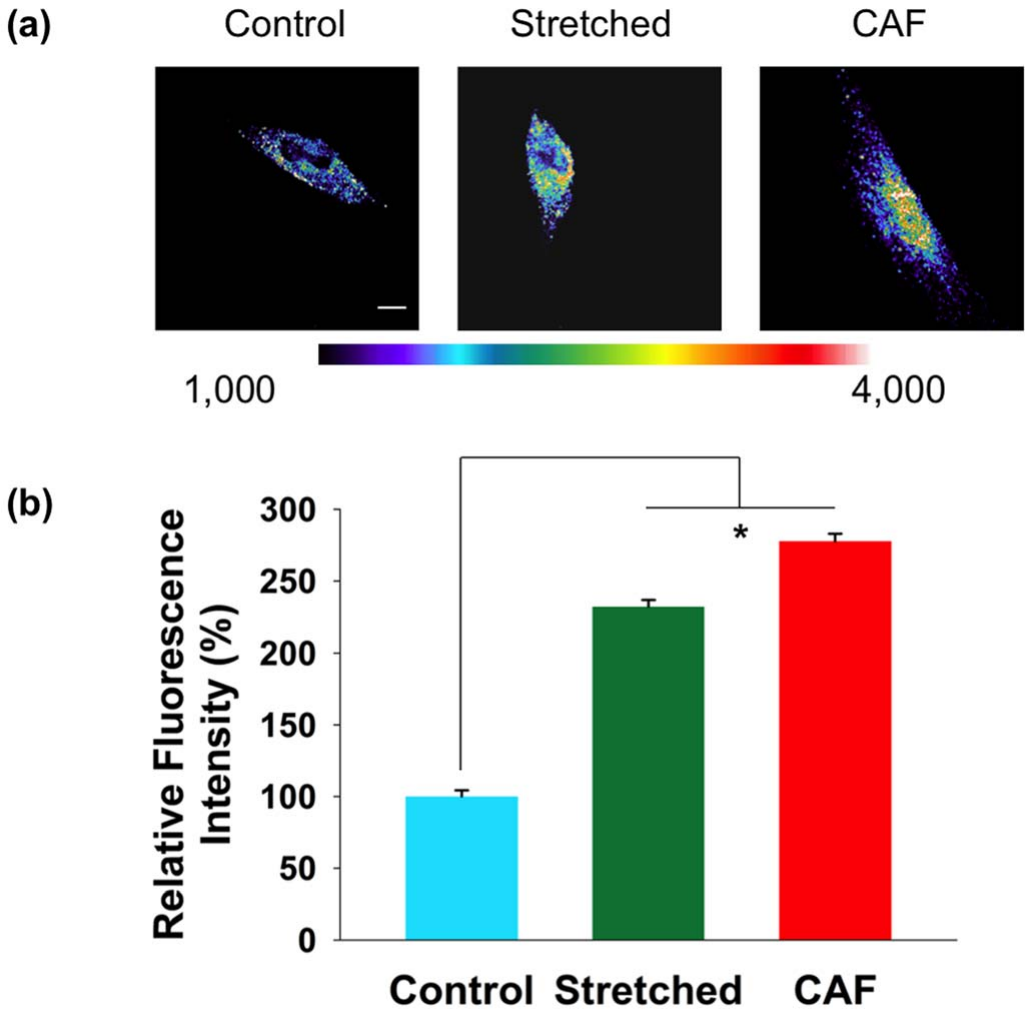
Stretched NAFs and CAFs show higher PDGFRα expression than unstretched NAFs. (a) Images are shown in pseudocolor which indicates the range of fluorescence intensities for the assigned color. CAFs and stretched NAFs have higher PDGFRα expression than unstretched NAFs. Scale bar is 50 μm. (b) Quantification of PDGFRα expression of unstretched NAFs, stretched NAFs, and CAFs. Error bar represents S.E.M from 40 cells from six individual experiments (*p < 0.005).

Our observations indicate that there is no general alignment of the fibroblasts with respect to the stretch direction; however, the deposited fibronectin does form an ordered structure around each fibroblast locally. These results strongly suggest that the applied mechanical stress, instead of simply introducing a direct biophysical response, triggers complex biochemical signaling cascades that affect how fibroblasts construct ECM. To confirm that this is indeed the case, we observed whether there is a biochemical change in stretched NAFs when compared with unstretched NAFs. Particularly, we examined whether stretched NAFs express PDGFRα, a molecule found in reactive tumor stroma^44^,^45^. We observed a significantly enhanced level of PDGFRα in stretched NAFs as compared with the unstretched controls (Fig. 5). The expression level of stretched NAFs is very similar to that of CAFs (Fig. 5), suggesting that the mechanical stress triggered a biochemical response consistent with activation of the NAFs. It is worth noting that even though many biochemical factor changes have been detected from stretched fibroblasts in the literature, we have not found a report showing altered PDGFRα expression in stretched fibroblasts. Therefore, our investigation discloses another biochemical factor that can be affected by mechanically stretching fibroblasts.

The activation of PDGF receptors may directly drive tumor growth via autocrine PDGF stimulation or through receptor mutations. However, it is also possible that the PDGF produced by cancer cells, which typically do not respond to PDGF themselves, may also affect nearby non-tumor cells such as fibroblasts through paracrine signaling^45^,^46^. As a result, the enhanced expression of PDGFRα observed in CAFs and stretched NAFs indicates that PDGF could be an important mediator in activating NAFs exposed to mechanical stress in the tumor microenvironment and in changing NAFs' biological behavior, such as altered fibronectin secretion/organization.

## Conclusion

In summary, we adapted and further improved a microfluidic platform that not only enables repeatable, consistent mechanical stretching of fibroblasts, but also allows for microscopic imaging of cancer cell migration in a co-culture system. We used this platform to study the effects of exerted stress on prostatic NAFs. Results indicate that mechanical force leads to the alignment of fibronectin secreted from stretched NAFs. Moreover, co-culture of stretched NAFs with SCC61 cells significantly enhanced the migration association index and directionality. These stretched NAF phenotypes are similar to those exhibited by CAFs but different from those observed in control cells, i.e., unstretched NAFs. Furthermore, we found that the expression of PDGFRα in stretched NAFs was increased to a level that is similar to that of CAFs, indicating that mechanical stress triggers complex biochemical reactions in stretched NAFs. These results provide insight into the important role that mechanical factors may play in NAF activation/CAF genesis. Although biochemical factors have been widely studied in the genesis of CAFs and their interactions with cancer cells, currently little is known about the role that biophysical factors, such as mechanical stress, play in this process. Therefore, the reported study, which examines the important function of mechanotransduction in the tumor microenvironment, should trigger more interest in the role of biophysical effects in the NAF activation/CAF genesis process.

## Methods

### Microfluidic Device Fabrication

The microfluidic stretcher platform consists of three primary components: a 2–3 mm thick polydimethylsiloxane (PDMS) (SYLGARD 184 Silicone Elastomer Kit, Dow Corning, Midland, MI) top layer, a ~10 μm thick spin-coated PDMS membrane, and a 100 μm thick thin-film PDMS bottom layer. Different protocols were used in the fabrication of each piece. For the thick PDMS top layer, traditional soft lithography methods were employed^47^,^48^. A 100 μm thick SU-8 (SU-8, Microchem Inc., Newton, MA) mold outlining the key channel features was fabricated on a silicon wafer using photolithography. PDMS mixed at a ratio of 10:1 (base polymer : curing agent) was poured on the SU-8 mold, degassed for ~1 hour, and cured in a 70°C oven for at least 2 hours. The ~10 μm thick PDMS membrane was prepared using a spin-coating process. A small amount of mixed, degassed PDMS was dropped on the treated side of a 3″ × 3″ plastic fluoropolymer coated polyester (PE) transfer sheet (Scotchpak™ 1002 Release Liner, 3M™, St. Paul, MN) that was taped to a Petri dish lid. Using a spin-coater, the PDMS was spun at 4000 RPM for ~1 minute, which was then cured for ~4 hours at 70°C. The 100 μm thick PDMS bottom layer was fabricated using the method described by Hsu et al^49^. A small amount of PDMS was spread thinly over an SU-8 mold and covered with a 3″ × 3″ fluoropolymer coated PE sheet. The mold-PDMS-PE sheet stack was then degassed for ~1 hour. A small plastic squeegee was used to push out any remaining air bubbles, after which 1 mm thick glass slides were pressed on top of the PE sheet to ensure clean contact between the PE sheet and the top of the SU-8 mold features. Five pounds of weights were added on top of the glass slides, and the whole package was placed in a 70°C oven for at least 4 hours to cure.

After completing the fabrication of each individual device layer, the three components were assembled into the final microfluidic stretcher platform. First, the thick PDMS top layer was attached to the spin-coated PDMS membrane using plasma bonding. After bonding, the PE transfer sheet was removed from the backside of the thin PDMS membrane layer, and holes were punched in the inlet/outlet of the central cell chamber channel and in the inlets of the two outer vacuum chamber channels. A 30-gauge needle was then used to make small tears in the PDMS membrane above the vacuum chamber regions. Next, the 100 µm thick PDMS bottom layer was plasma bonded to a 24 mm × 60 mm × 0.13 mm glass coverslip, after which the PE transfer sheet was removed. Finally, the two newly formed components were bonded together, with care taken to align the upper and lower channels. Inlet/outlet reservoirs and tubing were then attached to the device using mixed liquid PDMS as glue.

### Device Characterization Using Confocal Microscopy

To correlate the induced strain in the stretched membrane of the microfluidic device with the applied vacuum pressure, a Quorum WaveFX spinning disk confocal system equipped with a Nikon Eclipse Ti microscope was employed. Fluorescein isothiocyanate (FITC) was introduced into the upper chamber of the device, rendering the channel features clearly visible when excited by a 491 nm laser line. A stack of images (0.2 μm spacing between planes) was obtained at both the left and right edges of the cell culture chamber using a Plan Fluor 40X objective (NA 1.3) and a Hamamatsu ImagEM EM-CCD camera. Images were obtained from five separate stretcher devices with four different vacuum pressures applied: 0 kPa, 25 kPa, 50 kPa, and 75 kPa. An orthogonal view of each stack was reconstructed using ImageJ, which showcased exactly which plane of the stack represented the bottom of the channel/top of the membrane. The identified “top of the membrane” image in each stack was compared to the corresponding images at different vacuum pressures for a given stretcher device and position. The change in displacement of the outer edge of the membrane for increasing vacuum pressures was then measured. Strain (*ε*) was computed according to its definition as *ε = ΔL/L*, in which Δ*L* is the combined change in displacement of the left and right sides of the membrane and *L* is the original width of the membrane (1000 μm). The data for the five devices were averaged and the standard deviation calculated at each vacuum pressure.

### COMSOL Multiphysics™ Modeling

Finite element modeling (FEM) of the stretcher device was conducted using the Solid Mechanics module in COMSOL Multiphysics™. A two-dimensional model was first constructed, mimicking the dimensions of cell and vacuum chambers in the center of the device. Additional analysis using a three-dimensional representation of the device yielded results similar to the two-dimensional model, but required an increased computational load; thus, the two-dimensional model was deemed sufficient for use in this study. A density of 965 kg/m^3^ and a Poisson's ratio of 0.45 were chosen for PDMS. Of particular importance in this model is the value chosen for the Young's modulus. A range of Young's moduli for PDMS have been reported in the literature, varying from 12 kPa to 6.61 MPa^50^,^51^,^52^,^53^,^54^. The PDMS dimensions, processing conditions, and measurement techniques all appear to affect the determined moduli values in the literature. Thus, in order to more accurately represent the mechanical properties of PDMS in the model, the geometry was divided into three domains with different Young's moduli. In the bulk PDMS areas of the geometry, a modulus of 1.8 MPa was employed, which reflects the modulus reported for large slabs of PDMS^51^,^52^. However, for the side walls (75 μm thick) and the suspended membrane (10 μm thick), Young's modulus values of 1.14 MPa and 1.9 MPa were chosen, respectively. These values more accurately represent modulus values reported for thinner PDMS components that have been shown to be thickness-dependent^53^.

### NAF and CAF Cell Generation

Human prostatic CAFs and NAFs were prepared as previously described^3^. For this study, CAFs and NAFs from five separate patients were used to ensure consistency. Following preparation, cells were qualified using a tissue recombination bioassay to confirm that CAFs induced tumor formation from BPH1 cells and that NAFs did not elicit tumorigenesis. Aliquots of CAFs and NAFs were frozen in liquid nitrogen for later use. Cells were used at low passage (less than passage 10) to ensure proper function in ECM production and communication with cancer cells.

### Cell Maintenance and Co-Culture In Microfluidic Devices

Prostatic CAFs and NAFs were cultured and maintained in Roswell Park Memorial Institute medium (RPMI) 1640 with 10% fetal bovine serum (FBS) and penicillin–streptomycin as described previously^25^. Human head and neck cancer cells SCC61 were maintained in Dulbecco's Modified Eagle's/F12 Medium (Life technology, Carlsbad, CA) supplemented with 10% FBS and penicillin–streptomycin.

A week before co-culture, cancer cells were adapted to RPMI medium gradually. Cell growth and random migration assays were conducted to confirm that there was no significant change due to the medium switch. In each microfluidic device, ~5 × 10^3^ NAF cells suspended in 20 µL of RPMI medium were loaded in the inlet reservoir. Note that no special coating or surface activation of the PDMS membrane was performed before loading the cells. After incubating ~2 hours at 37°C to allow NAFs to attach to the suspended PDMS membrane, both inlets of the vacuum chambers on the stretcher device were attached to a vacuum line that had been inserted into the incubator. A vacuum pressure of 67.7 kPa was applied, and the device was incubated overnight at 37°C in a cell culture incubator with 5% CO_2_. When studying cancer cell migration in the presence of NAFs (both stretched and unstretched), ~5 × 10^3^ SCC61 cells labeled with CellTracker™ Green (Life technology, Carlsbad, CA) were suspended in 20 μL of RPMI and loaded on top of the NAFs growing inside the devices. The devices were then incubated overnight before being subjected to live-cell imaging. For studies using CAFs, cells were loaded into the inlet reservoir of a simple single-layer microfluidic cell culture chamber with dimensions similar to that of the stretcher platform. However, the cells were plated directly on a glass coverslip, not on a suspended PDMS membrane. Otherwise, tests involving CAFs were carried out identically to those using NAFs.

### Immunofluorescence Staining

Both CAFs and stretched/unstretched NAFs were fixed with 4% paraformaldehyde in 0.12 M sucrose in PBS for 15 minutes, then permeabilized with 0.2% Triton-X-100 in PBS for 5 minutes, and finally blocked in 10% normal goat serum. PDGFRα expression was probed with rabbit polyclonal antibody (D1E1E, Cell Signaling Technology, Danvers, MA), followed by Alexa Fluor® 555 goat anti-rabbit secondary antibody (Life Technology, Carlsbad, CA). For fibronectin staining, an antibody against fibronectin (610077, BD Bioscience, San Jose, CA) was used, followed by Alexa Fluor® 488 goat anti-mouse secondary antibody (Life Technology, Carlsbad, CA). Primary antibodies were diluted at 1:500 and secondary antibodies at 1:2000 in PBS with 0.2% Triton-X-100. Primary antibodies were incubated for 2 hours; secondary antibodies were incubated for 45 minutes. Excess, unbound antibody was completely washed out of the channel with PBS. The entire staining process was conducted at room temperature.

### Microscopy and Live-Cell Imaging In Microfluidic Devices

Both fixed and live-cell imaging were performed as described previously^55^. A Quorum WaveFX spinning disk confocal system was used that was equipped with a Nikon Eclipse Ti microscope, a Plan Fluor 20X objective (NA 0.75), a Plan Fluor 40X objective (NA 1.3), and a Hamamatsu ImagEM EM-CCD camera. To image fixed cells at 40X magnification, the top PDMS layer/membrane was cut and detached from the bottom layer, and the membrane was placed directly on a thin glass coverslip. This allowed for better images at higher magnification using the 40X objective. Images were acquired using MetaMorph software (Molecular Devices, Sunnyvale, CA). For migration assays, images were obtained every 5 minutes for 12 hours. Cells were maintained at 37°C using a temperature-controlled chamber (Live Cell Instrument, Seoul, Korea). CellTracker™ Green and a red fluorophore (Alexa Fluor® 555) were excited with 491 nm and 561 nm laser lines, respectively. Emission filters for these fluorophores were 470/24 and 525/50 (Semrock, Rochester, NY).

### Quantification of Fibronectin Alignment and Cancer Cell Migration

To quantify fibronectin alignment, the angles between individual fibronectin fibers were measured. A template with nine points evenly distributed in a round plane was designed as previously reported^56^. Confocal images of fibronectin were overlaid with the template and then subjected to analysis. For each of the nine points, the fiber closest to the point was identified, and then the intersection angle of the nearest crossing fiber was determined using ImageJ. A minimum of 50 angles were analyzed for each condition.

To quantify directional cancer cell migration, the total migration distance (T) for individual cells was measured from time-lapse images and the net (D) distance was calculated. The directionality index (D/T) was determined by taking the ratio of the net (D) to total distance migrated (T), as previously described^57^. Association was determined by calculating the angle σ between the axes of migration of cancer cells and fibroblasts. The association index was defined as the cosine of σ. An association index of 0 denotes cells that are migrating perpendicular to each other, whereas an index of 1 indicates that cells are migrating parallel to each other. Note that for all quantification methods, a Student's t-test was used to determine significant differences in the data.

## Author Contributions

M.A. and B.B. conducted the experiments. L.Y. performed the COMSOL modeling. O.F.C. prepared the cells. M.A., B.B., S.H., D.W. and D.L. contributed to data analysis and discussions. D.W. and D.L. supervised the project and M.A., B.B., D.W. and D.L. prepared the manuscript.

## Supplementary Material

Supplementary Information Migration of SCC61 cells co-cultured with unstretched NAFs

Supplementary Information Migration of SCC61 cells co-cultured with stretched NAFs
